# Use of tree-based models to identify subgroups and increase power to detect linkage to cardiovascular disease traits

**DOI:** 10.1186/1471-2156-4-S1-S66

**Published:** 2003-12-31

**Authors:** Tracy Jennifer Costello, Michael David Swartz, Mahyar Sabripour, Xiangjun Gu, Rishika Sharma, Carol Jean Etzel

**Affiliations:** 1 Department of Epidemiology, The University of Texas MD Anderson Cancer Center, Houston, Texas, 77030, USA; 2 Graduate School of Biomedical Sciences, The University of Texas Health Science Center, Houston, Texas, 77030, USA; 3 Department of Statistics, Rice University, Houston, Texas, 77005, USA; 4 Texas Academy of Mathematics and Science, Denton, Texas, 76203, USA

## Abstract

**Background:**

Our goal was to identify subgroups of sib pairs from the Framingham Heart Study data set that provided higher evidence of linkage to particular candidate regions for cardiovascular disease traits. The focus of this method is not to claim identification of significant linkage to a particular locus but to show that tree models can be used to identify subgroups for use in selected sib-pair sampling schemes.

**Results:**

We report results using a novel recursive partitioning procedure to identify subgroups of sib pairs with increased evidence of linkage to systolic blood pressure and other cardiovascular disease-related quantitative traits, using the Framingham Heart Study data set provided by the Genetic Analysis Workshop 13. This procedure uses a splitting rule based on Haseman-Elston regression that recursively partitions sib-pair data into homogeneous subgroups.

**Conclusions:**

Using this procedure, we identified a subgroup definition for use as a selected sib-pair sampling scheme. Using the characteristics that define the subgroup with higher evidence for linkage, we have identified an area of focus for further study.

## Background

Determining the underlying genetic basis in a complex disease challenges researchers due to the presence of multiple genes with modest effects, known and unknown environmental factors, and possible gene × gene and gene × environment interactions. In contrast, a homogeneous data set provides an optimal situation for identifying genes involved in a particular disease's etiology because confounding factors, either genetic or environmental, do not mask the power to detect linkage. Recursive partitioning or tree-based models (i.e., classification and regression trees [1,2]) have recently been proposed to identify homogeneous subgroups within a population to facilitate detection of quantitative trait loci (QTL) [3,4].

Cardiovascular disease (CVD) is a prime example of a complex disorder with numerous disease-related traits, determined by multiple genes and environmental factors, in which recursive partitioning can potentially aid in the detection and localization of the underlying genes. In this paper, we apply a recursive partitioning splitting rule developed by Shannon et al. [4] to assess genetic linkage in the Framingham Heart Study (FHS) data set provided by the Genetic Analysis Workshop 13 (GAW13). We are interested in augmenting sib-pair sampling by using this tree method to identify subgroups of sib pairs that show increased evidence of linkage of systolic blood pressure and other CVD-related quantitative traits to compelling candidate regions identified in recent literature. The splitting rule developed by Shannon et al. [4] has currently been developed for use one marker at a time. We apply an alternative pruning rule to determine homogeneous subgroups of sibling pairs from the FHS data set that show stronger evidence of linkage to particular markers.

## Methods

### Data description

The Framingham Heart Study data set consists of two longitudinal cohorts with information relating individuals across both sets. In the interest of brevity, we refer the reader to published descriptions of the data in this issue and in the literature [5,6]. Previous investigators used data from this study to assess linkage to CVD related traits and a number of recent publications present significant and suggestive linkage to markers throughout the genome. While genome-scan data were generously provided, the tree-based method we use is only capable of analysis of one marker at a time so we chose to focus on implicated regions on chromosomes 17 and 20. We selected chromosome 17 because of the conflicting results reported for the angiotensin-converting enzyme locus [7-11] and significant results reported for a marker at 67 cM [12] and between 60–65 cM [11]. Compelling evidence from three different studies from three different populations looking at systolic blood pressure and triglycerides guided us to chromosome 20 [13-15].

The recursive partitioning method developed by Shannon et al. [4] (described below) uses a splitting rule based on the Haseman-Elston [16] test (H-E) for linkage to form daughter nodes. Our response variable is the regression of squared pair difference of a trait onto sib-pair allele sharing, so we calculated identity by descent (*ibd*) for all loci on chromosomes 17 and 20 using the program ibdn (a modified version of ERPA [17]) and computed the squared pair difference for systolic blood pressure (*sqsbp*) and triglycerides (*sqtrig*). We constructed sib pair-specific explanatory variables for both continuous and categorical variables. For the continuous variables, we calculated the sib-pair averages: body mass index (*ave bmi*), weight (*ave wgt*), height (*ave hgt*), cholesterol (*ave chol*), HDL (*ave HDL*), triglycerides (*ave trig*), glucose (*ave gluc*), cigarettes per day (*ave cpd*), and number of drinks per day (*ave drink*)]. For the categorical variables we created sib-pair variates that count the number of sibs in the pair that fit a given condition: number of males (*psex*), number with high blood pressure (*phbp*), and number on hypertensive medication (*phrx*).

We combined information from Cohort 1, visit 12, with Cohort 2, visit 1, with the pedigree data. Since our primary outcome is systolic blood pressure, we required that individuals in the resulting sib-pair data set were between 18 and 75 years of age. Because BMI is a well-known risk factor, we wanted to minimize the effects of missing data on this variate. If an individual was missing height or weight during the visit of interest, we imputed the missing variable from the previous or subsequent visit. If we had information for both the prior and subsequent visit, we used the average.

### Recursive partitioning and pruning methodology

Shannon et al. [4] describe a splitting rule designed to subdivide sib-pair data into more homogeneous subgroups assumed to be linked to a common QTL using H-E regression. For each sib pair, this method requires the squared pair difference for the phenotype of interest and ibd status at marker(s) of interest to perform the H-E regression, plus covariates (discrete or continuous) measured for each pair to divide the data into subgroups. By design, the split creates two subgroups that are more homogeneous than the original group in an effort to compensate for the potential decrease in power leading to lack of detection of linkage. The Shannon splitting rule (reg; ) has been programmed as an extension to the RPART  function in S-PLUS [18]. Simulation studies described by Shannon et al. [4] show that this method can correctly identify subgroups in the data, thus improving the power to detect linkage while resulting in only a minor increase in false positives. Furthermore, this method was developed and tested to investigate increased evidence of linkage in a subgroup at a single marker locus without accounting for effects of other loci. However, we have not seen published applications of this method to real genetic data.

Tree-based models follow the same rule as all modeling procedures: a balance between parsimony and good fit. Therefore, large trees need to be pruned in order to make better interpretations. Traditional pruning rules [1] prune off branches based on the complexity parameter or misclassification error, but alternative pruning rules have also been proposed [2,19]. Of relevance is a description by Zhang and Singer [2] of a maximum χ^2 ^pruning rule for association tests in which the χ^2 ^statistic at each node is compared to a χ^2 ^critical value from a preset alpha level. If the χ^2 ^statistic at the node and subsequent nodes is not significant, the node is pruned. We propose an analogous pruning rule based on the H-E sib-pair linkage test to prune the trees we obtain using the previously described methodology. Since the H-E method is a simple linear regression with the null hypothesis that the regression slope equals zero versus an alternative hypothesis that the slope is negative, a *t*-statistic can be calculated for the regression coefficient at each node and compared to a predetermined one-sided significance level. If the *t*-value at the node and subsequent nodes is greater than the critical value then we prune the branch. We call this the *minT *pruning rule.

### Application to FHS/GAW13 data

We constructed regression trees, one per marker on chromosomes 17 and 20, using the data from the FHS with 10-fold cross-validation. We used H-E regression as our response, in which we regressed *sqsbp *or *sqtrig *onto *ibd *at each marker on chromosomes 17 and 20 and based splits on the constructed sib pair covariates. We utilized the Shannon method splitting rule and defined a set of parameters for the splits: 1) a node must have at least 150 sib pairs to be considered for a split, 2) at least 100 sib pairs were required for each terminal node, and 3) surrogate splits were permitted to allow for missingness. We set the complexity parameter equal to zero to allow each tree to grow to its full size and then pruned the tree back using our proposed *minT *pruning procedure to remove branches containing nodes with a *t*-value greater than -1.645 (alpha level of 5%).

## Results

**Figure 1:**
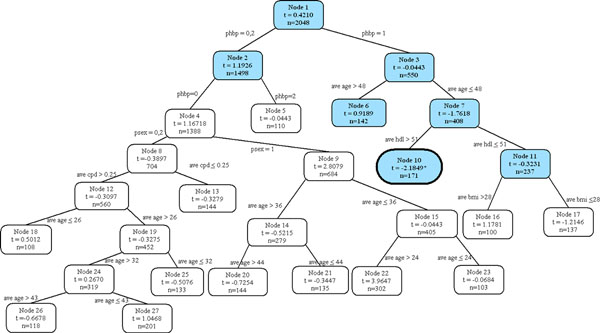
**Tree for D17S2180, overgrown (a) and pruned to optimal tree (b, shaded). **Each split is labeled with split criterion. Each node is labeled with node number and *t*-value obtained from the regression of squared sib-pair difference of systolic blood pressure on IBD for the subgroup of sib pairs at each node and the number of sib-pairs in the node.

**Figure 2:**
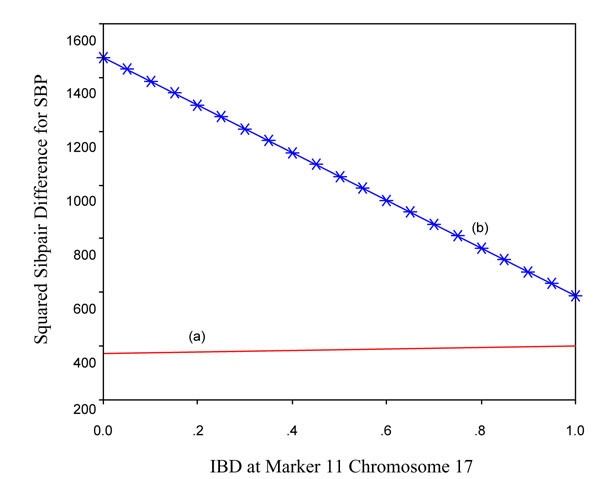
Haseman-Elston regression lines at D17S2180 for (a) all eligible sib pairs and (b) node 10 subgroup.

We conducted tree-based analysis on regions around candidate loci located on chromosomes 17 and 20. Figure 1 displays both the (a) full-grown tree and (b) pruned tree (shaded nodes) for the regression of sqsbp onto ibd at marker ATC6A06, also known as D17S2180. The full tree had a total of 13 terminal nodes. The t-value of 0.4210 at node 1 of the tree is from the H-E method using all of the sib pairs. Sib pairs were partitioned into groups based on concordance for high blood pressure (phbp), average age (ave age), average BMI (ave BMI), average HDL (ave HDL), average number of cigarettes per day (ave cpd) and number of males in the pair (psex). Node 10 had the minimum t-value of -2.1847, which represents the subgroup of 171 sib pairs who were discordant for phbp, had ave age less than 48 years and ave HDL greater than 51. No other branch of the tree had a t-value this extreme or less than the -1.645 critical value. Therefore, the tree was pruned so that node 10 was one of the four remaining terminal nodes. Figure 2 displays the H-E regression lines for (a) all sib pairs and (b) node 10 sib pairs. This is a meaningful result because at node 1, the slope of the H-E regression line for all the data shows no evidence of linkage, but the slope of the H-E regression line using the sib pairs that make up the node 10 subset show some evidence of linkage of systolic blood pressure with this marker. The consequence is that an investigator may use this subgroup definition to choose selected sib pair samples. This subgroup, instead of being chosen based on one trait of interest, is now defined on other meaningful covariates and related quantitative traits. Subsequent trees grown at flanking markers D17S1299 (at 62 cM), D17S1290 (82 cM), D17S2193 (89 cM), and D17S1301 (100 cM) on chromosome 17 identified similar subgroups of sib pairs that were discordant for phbp. At each of these markers, the first split of the tree is discordant high blood pressure and the second split is on the average age (about 45 years) of the sibling pair. Trees grown at the seven markers covering the region from 11 cM to 51 cM on chromosome 17, identified subgroups where neither sib pair had high blood pressure (trees not shown). The tree at markers D17S1293 (56 cM) and 044xg3 (117 cM) identified no subgroups (trees not shown).

We further constructed trees for the H-E regression of *sqsbp *onto *ibd *for the 11 markers on chromosome 20 (trees not shown). Sib pairs in which neither sib had high blood pressure showed higher evidence for linkage across the chromosome. For example, at marker D20S482 located at 12 cM, the node with increased evidence of linkage identified a subgroup of same-sex sib pairs for which neither sib had high blood pressure, average age of the sibs was less than 32 years, and on average they smoked less than 4 cigarettes per day and had average triglycerides less than 75. Xu et al. [15] reported suggestive evidence of linkage to this same region. However, their study was based on a population of Chinese sib pairs grouped by concordance/discordance of systolic blood pressure and they are continuing their analyses to take into account more covariates for subdividing the sib pairs in their study. At marker D20S604 (33 cM), we identified a subgroup in which the sibs that had average triglycerides greater than 60 displayed higher evidence of linkage to systolic blood pressure. Notably, Shearman et al. [13] reported linkage to this region (marker at 35 cM) with triglyceride levels.

We subsequently constructed trees for the H-E regression of *sqtrig *onto *ibd *for markers D20S604 (33 cM) and D20S470 (39 cM) on chromosome 20. Subgroups of sib pairs with average cholesterol greater than 196 and less than 140 were both identified from the trees. For marker D20S604, sib pairs who had average cholesterol greater than 196, where each sib drank less than one drink per day on average and who had an average HDL around 50 displayed highest linkage. A similar subgroup was identified in the tree for marker D20S604, except the average age of the sibs were greater than 43 years. The subgroup with the highest evidence for linkage for marker D20S604 contained sib pairs that had average cholesterol less than 140, each sib drank less than two drinks per day on average, and the average age of the sibs was greater than 46.

## Conclusions

Data mining methodologies have gained popularity in the field of genetic epidemiology. We applied the tree-based methods described above to identify subgroups in the FHS data set that provide more evidence for linkage. We do not claim identification of significant linkage to this region on chromosome 17 in FHS. Rather, we suggest that identification of pairs in subsequent studies with similar characteristics would enhance the power to detect linkage. Furthermore, Shannon et al. [4] proposed that this method has potential to uncover subgroups in the data that can ultimately enable the identification of complex disease loci and that this method can conceivably be extended to incorporate effects of multiple loci.

In this investigation, we constructed linkage trees at each marker on chromosomes 17 and 20. A more optimal approach would have been to construct multipoint regression trees. However, at the time of this investigation, the methodology had not been developed to handle this type of analysis. Therefore, we chose to complete our investigation one marker at a time. One might be concerned that investigating a tree for every marker represents a risk of multiple testing issues. Multiple testing is regarded as a gray area in genetic analysis, and also for tree modeling. However, our goal was not to determine true linkage to a trait, but to show that tree modeling can be used to refine selected sib-pair sampling. Subdividing sib pairs for linkage analysis is valuable because different subgroups of sib pairs are useful under different genetic models [15]. We also created a pruning rule specific to H-E regression because we were concerned that if one forms enough subgroups that one or more will have a linkage signal by chance. We observed fairly consistent subgroups of sib pairs identified by the trees at the markers adjacent to the 67 cM region of chromosome 17, which shows the strength and applicability of this method.

We are in the process of completing secondary analyses using SOLAR [20] to assess linkage using the families that include the 171 sib pairs from the subgroup identified from the tree-model of chromosome 17 marker D17S2180. We are interested in seeing if these families account for a significant proportion of the LOD score reported by Levy et al. [12]. It would have been preferable to use a secondary data set for the follow-up analysis. We acknowledge that using the same data set to first identify a compelling subgroup and then to detect linkage overuses the data. However, at the time of this manuscript preparation, a second data set was unavailable for follow-up analysis.
